# Suppression of OCT-1 in Metastatic Breast Cancer Cells Reduces Tumor Metastatic Potential, Hypoxia Resistance, and Drug Resistance

**DOI:** 10.3390/life12091435

**Published:** 2022-09-15

**Authors:** Alexander G. Stepchenko, Elizaveta V. Bulavkina, Tatiana N. Portseva, Sofia G. Georgieva, Elizaveta V. Pankratova

**Affiliations:** 1 Engelhardt Institute of Molecular Biology, Russian Academy of Sciences, Vavilov Str., 32, 119991 Moscow, Russia; 2 Center for Precision Genome Editing and Genetic Technologies for Biomedicine, Engelhardt Institute of Molecular Biology, Russian Academy of Sciences, 119991 Moscow, Russia

**Keywords:** breast cancer, therapeutic targets, OCT-1 transcription factor, metastasis, drug resistance

## Abstract

OCT-1/POU2F1 is a ubiquitously expressed transcription factor. Its expression starts at the earliest stage of embryonic development. OCT-1 controls genes involved in the regulation of differentiation, proliferation, cell metabolism, and aging. High levels of OCT-1 transcription factor in tumor cells correlate with tumor malignancy and resistance to antitumor therapy. Here, we report that suppression of OCT-1 in breast cancer cells reduces their metastatic potential and drug resistance. OCT-1 knockdown in the MDA-MB231 breast cancer cells leads to a fivefold decrease (*p* < 0.01) in cell migration rates in the Boyden chamber. A decrease in the transcription levels of human invasion signature (HIS) genes (*ARHGDIB*, *CAPZA2*, *PHACTR2*, *CDC42*, *XRCC5,* and *CAV1*) has been also demonstrated by real-time PCR, with high expression of these genes being a hallmark of actively metastasizing breast cancer cells. Transcriptional activity of ATF6 response elements is significantly reduced in the cell lines with decreased OCT-1 expression, which results in lower levels of adaptive EPR stress response. OCT-1 knockdown more than two times increases the MDA-MB231 cell death rate in hypoxia and significantly increases the doxorubicin or docetaxel-treated MDA-MB231 cell death rate. Our findings indicate that OCT-1 may be an important therapeutic target and its selective inhibition may have significant therapeutic effects and may improve prognosis in breast cancer patients.

## 1. Introduction

Breast cancer is considered to be among the most common malignant tumors in women in developed countries, with metastases being the most frequent cause of mortality in breast cancer patients. Personalized medicine and the development of prognostic molecular profiling-based tests stimulated intensive research aimed at the identification of genetic determinants of cancer in the previous ten years. This research work led to the discovery of gene sets or gene signatures, the expression of which in primary tumors is associated with poor prognosis in cancer patients. 

The *POU2F1* (*POU2F1* Class 2 Homeobox I) gene encoding the OCT-1/POU2F1 transcription factor (further referred to as OCT-1) belonging to the POU transcription factor family was shown to be among these genes. This protein is known to be ubiquitously expressed in the human organism [1,2]. OCT-1 has diverse roles in transcription regulation and is essential for normal cell functioning as it controls genes which regulate cell proliferation, migration, differentiation, adhesion, cell metabolism, and aging [2,3,4,5,6,7,8,9]. OCT-1 may act as both transcription activator and repressor and is considered to be a multifunctional regulator with the ability to broadly change gene expression patterns. OCT-1 can form a transcription–repression complex by anchoring chromatin loops to the nuclear membrane. Genes associated with cell aging, including the collagenase gene, appear to be derepressed when OCT-1 protein dissociates from the nuclear peripheral structure [6]. Binding of OCT-1 to the peripheral nucleus via lamin B1 may be a mechanism by which the nuclear structure can regulate gene expression and contribute to cellular response to stress, growth, and aging [7].

Transcription of the *POU2F1* gene is regulated by two different promoters, namely, the ubiquitous promoter and the tissue-specific promoter [2], which results in the transcription of two alternative 5′-exons, and the presence of two different N-terminal regions in the OCT-1 protein. OCT-1 exists in several isoforms, with the OCT-1A isoform being ubiquitously expressed [2,3] and dominating in most human somatic cells including normal and tumor breast cells. This transcription factor undergoes post-translational modifications and was shown to be involved in the interaction with at least 60 cellular proteins with canonical and a number of non-canonical DNA-binding sites [1,2,3,4].

Recently, a statistically significant correlation between high OCT-1 expression levels in tumor cells and tumor malignancy rates has been demonstrated [5,8,9,10]. This protein expression level is a marker of very poor prognosis in gastric cancer and several other cancers, providing more accurate prognostic information that AJCC. OCT-1 is currently thought to be a promising prognostic marker for several types of epithelial cancer, including breast cancer [1,11,12,13,14]. The analysis of OCT-1 protein content in the primary tissues of breast cancer patients revealed higher OCT-1 levels compared to normal tissues [10]. Other studies have demonstrated OCT-1 enrichment in the target OCT-1 sites in breast cancer and other malignant tumors, OCT-1 being also a cancer stem cell determinant [15,16,17,18,19,20,21,22,23,24].

Interactome network analysis in breast cancer has identified OCT-1 as a member of a protein set contributing to malignancy [20]. Increased OCT-1 level in breast cancer cells shifts cell metabolism towards glycolysis, for BRCA1 governs OCT-1 ubiquitination and degradation thereby promoting oxidative metabolism and restricting OCT-1 oncogenicity [25]. In breast cancer cells, the increase in OCT-1 levels inhibits the expression of the circadian clock gene *Period2* (*PER2*), which is a tumor suppressor [17]. Therefore, it can be seen that high levels of OCT-1 expression in breast tumors disturb many cellular processes, with disorders resulting from it possibly contributing to malignization, higher tumor aggressiveness, and higher tumor resistance to chemotherapeutics.

To test the hypothesis that OCT-1 may be an important therapeutic target in breast cancer, we analyzed cell migration, growth rate, response to endoplasmic reticulum stress, and cell survival under hypoxia conditions following a reduction in the total OCT-1 or OCT-1A isoform expression levels in a triple-negative breast cancer cell line (MDA-MB231). Triple-negative breast cancer is a group of aggressive cancers with poor prognosis due to high metastasis ability, recurrence rates, and chemotherapy resistance. New approaches to reducing malignancy and chemoresistance of this type of cancer need to be developed. MDA-MB231 cells are the best-studied triple-negative breast cancer cell line (estrogen receptor (ER)-, progesterone receptor (PR)-, HER2-). The results obtained in the present work suggest that downregulation of OCT-1 expression significantly reduces migration, hypoxia resistance, response to endoplasmic reticulum stress, and drug resistance of MDA-MB231 cells and thus may be taken advantage of in developing strategies to reduce triple-negative breast cancer malignancy and chemoresistance.

The results obtained in the present work suggest that a decrease in the intracellular OCT-1 levels may alleviate the destructive effects exerted by cellular metabolism deregulation. We have demonstrated that decreased OCT-1 levels have a significant inhibitory effect on cell migration, hypoxia, and ER stress resistance as well as on the resistance to chemotherapeutics in the MDA-MB231 cells. We have also studied the effects of OCT-1 knockdown on HIS (human invasion signature) gene expression [26]. We have shown that OCT-1 knockdown suppresses the expression of the *ARHGDIB*, *CAPZA2*, *PHACTR2*, *CDC42*, *XRCC5,* and *CAV1* genes, which is known to be increased in actively metastasizing breast cancer cells. At the same time, OCT-1 knockdown had no statistically significant effect on the proliferation of MDA-MB231 cells. Our findings indicate that selective inhibition of total OCT-1 or its OCT-1A isoform might have a substantial therapeutic effect in breast cancer cells and improve disease prognosis in breast cancer patients.

## 2. Materials and Methods

Cell lines. Human breast cancer epithelial cell line MDA-MB231 (breast adenocarcinoma) (estrogen receptor (ER)-, progesterone receptor (PR)-, HER2-) was used in the work. The MDA-MB231 cell line was provided by Sigma-Aldrich Corp. Cells were grown in the complete DMEM (GIBCO, Thermo Fisher Scientific, Waltham, MA, USA) medium containing 10% fetal bovine serum (FBS; HyClone, UT, USA), 100 U/mL of penicillin and 100 μg/mL of streptomycin.

OCT-1 gene knockdown. RNA interference was used to knock down the OCT-1 gene. The lentivirus pGPV.ma4 vector was used to obtain the following expression constructs encoding short hairpin RNAs (shRNAs): control (empty vector with scrambled shRNA), OCT-1-shRNA (shRNA to knockdown total mRNA encoding all OCT-1 isoforms), and OCT-1A-shRNA (shRNA to knockdown mRNA encoding the OCT-1A isoform), which were used to transfect HEK-293 cells and produce the corresponding lentivirus stocks. The MDA-MB231 cells (105 cells) were transduced with the obtained lentivirus particles. After puromycin selection (1 μg/mL, 4 days), three MDA-MB231 lines including the two lines with the stable knockdown of total OCT-1 and the OCT-1A isoform and the control line (scrambled), were obtained. Puromicin (0.5 μg/mL) was used to maintain stably transformed cells and was removed from the medium 3 days before the experiment.

shRNA for total OCT-1 mRNA knockdown (OCT-1-shRNA):

5′-gatccGCCAAGACCTTCAAACAAATTCAAGAGATTTGTTTGAAGGTCTTGGCTTTTTTg-3′.

shRNA for OCT-1A mRNA knockdown (OCT-1A-shRNA):

5′-gatccACGGAGGAGCAGCGAGTCATTCAAGAGATGACTCGCTGCTCCTCCGTTTTTTTg-3′.

Scrambled shRNA, a nucleotide sequence showing no homology to any human RNA (control) was used as the negative control: 

5′-gatccGCAAAAATTCTCCGAACGTGTTCAAGAGACACGTTCGGAGAATTTTTGTTTTTTg-3′.

OCT-1 expression levels were measured by real-time qPCR and immunoblotting. The following primary and secondary antibodies were used in the work: rabbit polyclonal anti-OCT-1 antibodies previously obtained in our laboratory [2], rabbit polyclonal anti-OCT-1 antibodies (Abcam, Ab66132, Cambridge, Great Britain), mouse monoclonal anti-Lamin B1 antibodies (sc-377000; SantaCruse), rabbit polyclonal anti-β-actin antibodies (Abcam, Ab6276), goat anti-mouse HRP-conjugated antibodies (Jackson ImmunoResearch, 115-035-174), goat anti-rabbit HRP-conjugated antibodies (Jackson ImmunoResearch, 111-035-144). 

### 2.1. RNA Isolation and qRT-PCR Analysis

RNA was isolated from cells using Trizol. mRNA levels were measured by reverse transcription and real-time PCR and normalized to glucuronidase B (GUS)-encoding mRNA levels. Reverse transcription was performed using 2 µg of total RNA and the Maxima First Strand cDNA Synthesis Kit for RT-qPCR (Thermo Scientific) according to the manufacturer’s recommendations. cDNA was synthesized using mixed oligo(dT)18 and random hexamer primers. Primers used were as follows: OCT-1A-F 5′-tattcaaatggcggacgga-3′; OCT-1A-R 5′-gtttctgacggattgttcattc-3′;

ARHGDIB-F 5′-ctcggcctgaggagtatgag-3′; ARHGDIB-R 5′-gtggtcttgcttgtcatcgt-3′;

CAPZA2-F 5′-tacgtcgacagttgccagtt-3′; CAPZA2-R 5′-tctgcatctctttgccaatc-3′

CDC42-F 5′-tacgaccgctgagttatcca-3′; CDC42-R 5′-atctcaggcacccacttttc-3′;

XRCC5-F 5′-cctgaaagcccttcaagaga-3′; XRCC5-R 5′-agaggcttcctctttggtga-3′;

PHACTR2-F 5′-agaggcccacaactgaagaa-3′; PHACTR2-R 5′-ggctgagctttctgctgagt-3′;

CAV1-F 5′-cgtctgtgacccactctttg-3′; CAV1-R 5′-gatgcggacattgctgaata-3′;

GUS-F 5′-cgtggttggagagctcatttgga-3′; GUS-R 5′-attccccagcactctcgtcggt-3′.

Real-time qPCR was performed in the LightCycler96 thermal cycler (Roche, Basel, Switzerland). The standard reaction mixture (25 μL) contained the corresponding primer pairs, cDNA equivalent of 50 ng of total RNA, and qPCRmix-HS SYBR Master Mix (Evrogen, Russia). Real-time PCR conditions were as follows: 55 °C for 2 min, 95 °C for 5 min, followed by the 40 cycles of 95 °C for 10 s, and 59 °C for 30 s (signal acquisition temperature). In each case, the measurements were carried out in at least three replicates, and mean values were calculated. The obtained raw data was processed with the aid of the LightCycler96 Instrument software. qRT-PCR data were processed by calculating ∆Ct.

### 2.2. Western Blot Analysis

Cell protein extracts (10 μg) mixed with the DTT-containing Laemmli loading buffer were incubated at 40 °C for 10 min, applied to 8% SDS-PAGE, and then transferred onto the nitrocellulose membrane (GE Healthcare) in 25 mM Tris, 192 mM glycine, and 20% methanol, with subsequent membrane blocking in 5% nonfat milk in PBS for 1 h at room temperature. Antibodies against total OCT-1, OCT-1A isoform, Lamin B, or beta-actin were used in the Western blot assay. Lamin B or beta-actin was used as a loading control for subsequent normalization. Membranes were probed with primary antibodies in PBS supplemented with 0.1% Tween-20 (PBS-T) overnight and then washed 3 times for 20 min with PBS-T and incubated for 1 h at RT with the anti-mouse HRP antibodies (Santa-Cruz Biotechnology, sc-2005, 1:5000) or anti-rabbit HRP antibodies (Santa-Cruz Biotechnology, sc-2005, 1:5.000). After four washing steps with PBS-T, signal detection was performed according to the standard protocol using the ECL-reagent (GE Helthcare). Western blot assay results were visualized, and the obtained signal was quantitatively assessed using the ChemiDoc MP Imaging System (Bio-Rad) with the aid of the Bio-Rad Image Lab software. In each experiment, the measurements were made in triplicates, and mean values were calculated.

### 2.3. Cell Migration

The migratory activity of cells was determined by Transwell (Corning) with direct calculation of the number of migrated cells. Cell migration time was set as 48 h. Cells were grown in the DMEM medium in the Transwell chamber in the provided 24-well plates in four replicates for each cell line (1 × 105 cells/well). The upper and lower chamber compartments were separated from each other with a polycarbonate membrane 6.5 mm in diameter with the pore size of 8 μm. The upper chamber contained DMEM medium without serum, and the lower chamber contained DMEM medium with 10% fetal bovine serum (chemoattractant). Simultaneously, the same number of cells were grown in an ordinary 24-well plate in the DMEM medium containing 10% fetal bovine serum (control wells). After 48 h of growth, cells were detached using trypsin, and the number of cells which migrated via the Boyden chamber membrane and the number of cells in the control wells were calculated, and the percentage of migrated cells was determined relative to the number of cells in the control well, and mean values were calculated.

### 2.4. Growth Rate Calculation

Anti-OCT-1, anti-A, and control MDA-MB231 cells were placed into the 24-well plates in the amount of 50,000 cells per well in four replicates for each cell line. Cells were cultured in the complete DMEM medium, and every day over the course of five days, cells were detached with trypsin in four wells for each line, and the number of cells in these wells was calculated. Three biologically independent experiments were carried out, and mean values were calculated.

### 2.5. Cell Stress

MDA-MB231 cells were cultured in the complete DMEM medium. ER stress was induced by culturing cells in the presence of 1 µg/mL of tunicamycin for 24 h. To study the role of total OCT-1 or OCT-1A isoform knockdown in cell response to hypoxia or doxorubicin (Sigma-Aldrich) and docetaxel (Sandoz), cells were placed into the 96-well plates containing the complete DMEM medium in the number of 30,000 cells per well in five replicates. Then, 24 h later, cells were cultured in the complete DMEM medium at 370 °C with 0.5% O_2_ (hypoxia) for 24 h or in the presence of 1 µg/mL of doxorubicin for 48 h. Cell viability under hypoxia or in the presence of doxorubicin was measured using the CytoTox-Glo Cytotoxicity Assay (Promega). The percentage of live cells relative to the total number of cells and mean values were calculated.

### 2.6. Constructs Transfection and Dual Luciferase Assay

To study the role of total OCT-1 or OCT-1A isoform knockdown in cell response to ER stress, the pGL4-Firefly Luciferase Reporter Vectors with Cellular Stress Response Elements (Promega)—pGL4.39[luc2P/ATF6 RE/Hygro] were used. MDA-MB231-antiPOU, MDA-MB231-antiA, and MDA-MB231-control cell lines were transfected with the plasmid together with the pRL-Tk plasmid (Promega) using the TransFast reagent (Promega) according to the manufacturer’s recommendations. Cells were placed into the 96-well plates in the amount of 30,000 cells per well in five replicates one day prior to transfection. After transfection, cells were cultured in the complete DMEM medium at 370 °C with 5% CO_2_ for 24 h. After the end of the culturing period, cells were subjected to ER stress for 24 h as described above. Then, Dual Luciferase assay was carried out using the Dual-Glo Luciferase Kit (Promega). Luciferase signal was detected using the GloMax96 device with 0.5 s integration time, and mean values were calculated.

### 2.7. Statistics

Statistical analysis was performed using the GraphPad software (GraphPad Software, San Diego, CA, USA). The unpaired Student’s *t*-test was used to obtain *p*-values. Error bars represent S.E.M. (* *p* < 0.05, and ** *p* < 0.01).

## 3. Results

### 3.1. A Decrease in OCT-1 Expression Levels in the MDA-MB231 Cells

To study the functional activity and the effects of different OCT-1 concentrations in breast cancer, we suppressed OCT-1 protein expression in the MDA-MB231 triple-negative breast cancer cells. Three expression constructs for shRNA production were obtained on the basis of the pGPV.ma4 lentivirus vector, including the control construct (empty vector), the (OCT-1-shRNA) construct to produce the shRNA to knock down total mRNA encoding all OCT-1 isoforms, and the (OCT-1A-shRNA) construct to produce the shRNA to knock down the mRNA encoding the OCT-1A isoform, which is the ubiquitous isoform [2] produced at high levels in the MDA-MB231 cells.

**Figure 1:**
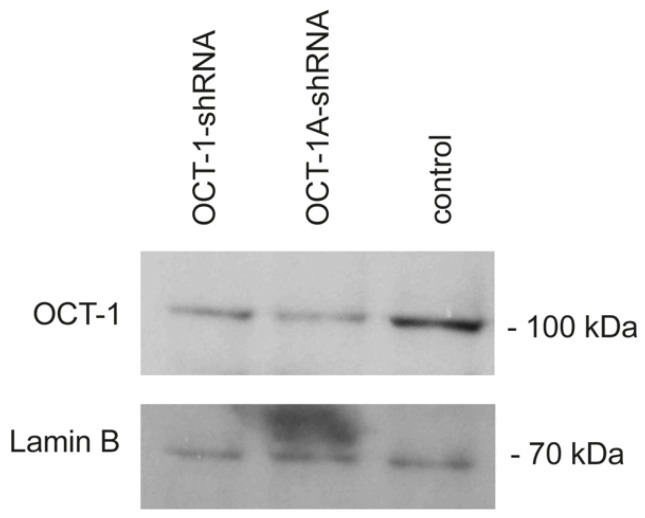
Suppression of total OCT-1 protein and its OCT-1A isoform expression in breast adenocarcinoma MDA-MB231 cells. Western blot with the antibodies against total OCT-1. MDA-OCT-1-shRNA— cells transduced with the lentivirus construct encoding the shRNA targeting total OCT-1 protein; MDA-OCT-1A-shRNA—cells transduced with the lentivirus construct encoding the shRNA targeting the OCT-1A isoform; MDA-control—cells transduced with the control “empty” lentivirus construct. Ten μg of cell extract per lane. Lamin B1 was used as a loading control. Whole-size Western blots image and mRNA expression levels are provided in Supplementary Figure S1.

As a result of MDA-MB231 transduction with the corresponding lentiviruses and subsequent puromycin selection, we obtained two cell lines in which total OCT-1 and the OCT-1A isoform were stably knocked down, along with the control cell line. To verify knockdown results, Western blot was used, which revealed an about sixfold decrease in OCT-1 protein expression (Figure 1). The obtained MDA-MB231 cell lines with OCT-1 knockdown were used in the proliferation and migration assays and in hypoxia and endoplasmic reticulum stress response tests as well as in the anticancer drug resistance tests.

### 3.2. Knockdown of the Total OCT-1 or Its OCT-1A Isoform Inhibits Breast Cancer Cell Migration and Decreases the Expression of Human Invasion and Metastasis Genes but Does Not Affect Cell Growth Rates

We used the MDA-MB231 tumor cells as a model to study the role of OCT-1 in breast cancer metastasis. This breast adenocarcinoma cell line is broadly used by the scientific community to study metastasis in vivo because of its ability to spontaneously produce orthotopic tumors when implanted into mouse tumor models and metastasize to other organs.

**Figure 2:**
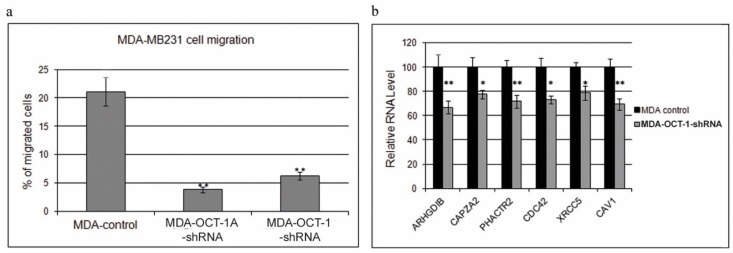
The effects of OCT-1 and OCT-1A isoform expression inhibition by knockdown on breast cancer MDA-MB231 cell migration. (**a**) OCT-1 knockdown inhibits cell migration in the Boyden chamber. MDA-MB231 line cells: MDA-control—cells transduced with the control “empty” lentivirus construct; MDA-OCT-1-shRNA—cells transduced with the lentivirus construct encoding the shRNA targeting total OCT-1 (OCT-1) protein; MDA-OCT-1A-shRNA—cells transduced with the lentivirus construct encoding the shRNA targeting the OCT-1A isoform. Cells were placed into the 24-well plates in four replicates for each cell line. The percentage of migrated cells was determined relative to the number of cells in the control well. (**b**) The transcription level of genes responsible for MDA-MB231 migration in the OCT-1 knockdown and control MDA-MB231 cells. Gene transcription was analyzed using qPCR, with the obtained results being expressed as percentages with mRNA level in the control line taken as 100%. Error bars represent S.E.M. (* *p* < 0.05, and ** *p* < 0.01).

We analyzed the effects of decreased OCT-1 and OCT-1A isoform levels on the migratory activity of MDA-MB231 cells. Cell migration was studied using the Boyden chamber by directly calculating the number of migrated cells. Migration time was set as 48 h. A significant decrease in the MDA-MB231 cell migration activity was observed in the cells with the OCT-1 or OCT-1A isoform knockdown. OCT-1 knockdown led to a 80% decrease in cell migration rates (*p* < 0.01) (Figure 2a).

The previous studies of breast cancer metastasis mechanisms have identified a number of invasion and metastasis (HIS) genes which expression is increased in cells with high migratory activity in both MDA-MB231 and breast tumor cell populations [27]. The changes in HIS gene expression are observed at the early stages of the metastatic cascade, being characteristic of primary tumor cell invasion and migration. In the present work, we analyzed the expression of HIS genes in the control and OCT-1 knockdown MDA-MB231 cells. OCT-1 knockdown led to a decrease in the expression levels of six [27] metastasizing cell marker genes (*ARHGDIB*, *CDC42*, CAZPA2, *XRCC5*, *PHACTR2,* and *CAV1*) (Figure 2b).

The data presented in Figure 2b suggest that OCT-1 plays part in downregulating the expression of certain genes responsible for MDA-MB-231 cell migration. It can be suggested that a simultaneous decrease in their expression has a synergetic effect and greatly reduces the migration ability of the MDA-MB231 tumor cells.

**Figure 3:**
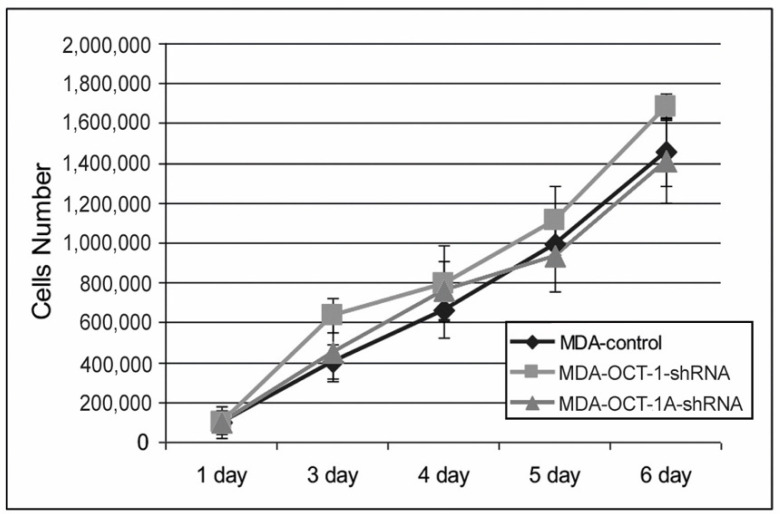
Downregulation of OCT-1 in MDA-MB231 cells does not affect cell growth rates. MDA-OCT-1-shRNA, MDA-Oct-1A-shRNA, and control MDA-MB231 cells were placed into the 24-well plates in four replicates for each cell line and the number of cells in these wells was calculated every day. Three biologically independent experiments were carried out, and mean values were calculated. Error bars represent S.E.M.

The analysis of cell division rates has demonstrated that decreased expression of either total OCT-1 or its OCT-1A isoform has no effect on the growth rate of breast cancer MDA-MB-231 cells (Figure 3). Our data indicate that OCT-1 most probably affects metastatic disease progression rather than primary tumor growth.

### 3.3. A Decrease in the OCT-1 and Its OCT-1A Isoform Expression Increases Hypoxia-Associated MDA-MB231 Cell Death

Tumor cells can adapt to hypoxia and attain more aggressive therapy-resistant cancer phenotypes as a result. The effects of OCT-1 expression inhibition on cancer cell resistance to hypoxia were studied using the MDA-MB231 cells with decreased expression of OCT-1 and OCT-1A isoforms. Cells were incubated under “severe” hypoxia conditions (0.5% O_2_) for 48 h, and the percent of dead cells was determined using the CytoTox-Glo Assay (Promega).

**Figure 4:**
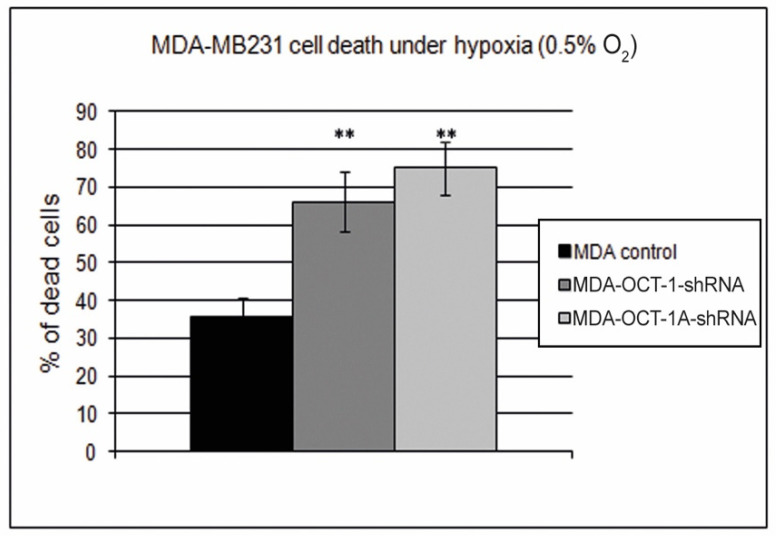
OCT-1 and its isoform OCT-1A knockdown reduces breast cancer cell survivability in hypoxia. Histogram representing the percentage of dead cells under hypoxic conditions. MDA-MB231 lines: MDA-control, MDA-OCT-1-shRNA, and MDA-Oct-1A-shRNA. Three biologically independent experiments were carried out, and mean values were calculated. Error bars represent S.E.M. (** *p* < 0.01).

The results of this experiment have shown that a decrease in OCT-1 expression in the MDA-MB231 cells leads to a considerable drop in the resistance of breast cancer cells to hypoxia. In the case of OCT-1 hypoexpression, cell death levels under hypoxia conditions appeared to be two times increased (Figure 4).

### 3.4. A Decrease in OCT-1 and OCT-1A Isoform Expression Inhibits Endoplasmic Reticulum Stress Response

Compensated endoplasmic reticulum (ER) stress induces the adaptive (protective) unfolded protein response (UPR) which works to maintain cell homeostasis. Simultaneous activation of PERK, ATF6, and IREI enables the protective response to ER stress making cells resistant and able to survive under this type of stress. In contrast, the suppression of ATF6 or IRE1 activation leads to decompensated EPR stress and cell death.

We have demonstrated that a decrease in the OCT-1 levels in tumor cells leads to a decrease in the transcription activity of the ATF6 response elements present in the regulatory region of the ATP6 target genes. ATF6 is a transcription factor which activates the target genes for the unfolded protein response (UPR) during endoplasmic reticulum (ER) stress. It functions as an ER stress sensor/transducer and following ER stress-induced proteolysis acts as a nuclear transcription factor exerting its effects via the cis-acting ER stress response element (ERSE), which is present in the promoters of the genes encoding ER chaperones. 

To study the role of total OCT-1 or OCT-1A isoform knockdown in cell response to ER stress, the pGL4-Firefly Luciferase Reporter Vectors containing several copies of the ATF6 response element sequence in the promoter—pGL4.39[luc2P/ATF6 RE/Hygro] were used. MDA-MB231-OCT-1-shRNA, MDA-MB231-OCT-1A-shRNA, and MDA-MB231-control cell lines were transfected with this vector together with the pRL-Tk plasmid. Cells were subjected to ER stress for 24 h in the presence of 1 µg/mL of tunicamycin. Dual Luciferase assay was further carried out using the Dual-Glo Luciferase Kit.

**Figure 5:**
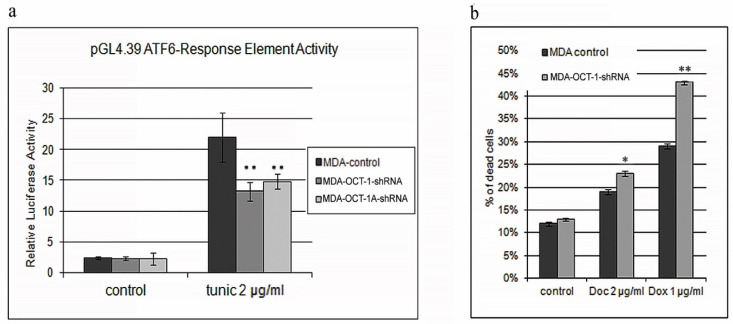
Suppression of OCT-1 leads to a decrease in the defence response. (**a**). The activity of the ATF6 response element-containing promoter under ER stress conditions caused by tunicamycin in the MDA-MB231 tumor cells: MDA-control, MDA-OCT-1-shRNA, MDA-OCT-1A-shRNA. (**b**). The effect of decreased OCT-1 levels in the MDA-MB231 cells on cell survivability following the treatment with chemotherapeutics. Histogram representing the percentage of dead cells following Doxorubicin and docetaxel treatment for 24 h. MDA-MB231 lines: MDA-control, MDA-anti-OCT-1. Three biologically independent experiments were carried out, and mean values were calculated. Error bars represent S.E.M. (* *p* < 0.05, and ** *p* < 0.01).

The results of this test revealed that the transcription activity of ATF6 response elements is significantly reduced in the cell lines with decreased OCT-1 expression (Figure 5a). It is important to note that inability to compensate EPR stress induces programmed cell death [28]. Thus, it may be assumed that a decrease in the cellular OCT-1 levels leads to a lower adaptive EPR stress response.

The activation of the endoplasmic reticulum stress protection is observed in different malignant tumors rendering them more resistant to chemotherapeutic drugs. To study the effects of decreased OCT-1 expression on breast cancer cell resistance to chemotherapeutics, MDA-MB231 cells with decreased OCT-1 expression were treated with Doxorubicin or docetaxel in the concentration of 1 µg/mL or 2 µg/mL, respectively, for 24 h. The results of this test demonstrated that reduced OCT-1 expression causes a considerable increase in Doxorubicin-treated MDA-MB231 cell death rates and a smaller but still significant increase in docetaxel-treated cell death (Figure 5b). Suppression of OCT-1 expression leads to higher apoptosis levels following the treatment with chemotherapeutic drugs (Figure S2).

## 4. Discussion

Pro-oncogenic properties have been noted for OCT-1 both in epithelial and non-epithelial tumors [1,5,6,7,8,9,16,21,26,29,30,31,32,33,34,35,36,37,38]. To test the hypothesis that OCT-1 transcription factor may be an important therapeutic target in breast cancer, we analyzed migration, growth rate, and survivability of the MDA-MB231 cells with decreased OCT-1 expression levels. We demonstrated that OCT-1 is the gene which controls the critical stages of breast cancer development, including metastasis and tumor cell resistance to hypoxia and EPR stress. Our study revealed a significant inhibitory effect of OCT-1 knockdown on migration, resistance to hypoxia and ER stress and chemotherapeutics resistance of the MDA-MB231 triple-negative breast cancer cells.

Currently, the data on migration and invasion, the two most important and potentially growth-independent initial stages of the metastatic cascade, still remain very limited. Tumors are known to have a very heterogeneous nature with not all cells in a tumor being migrating and invasive [39]. Using vital multiphoton microscopy, it was visualized that only a small portion of tumor cells within a primary tumor in the breast tumors of mice and rats have the ability to invade and migrate [40,41]. The work [26] reported expression profiles of genes specific for invasion and migration of human primary breast cancer cells and MDA-MB231 cells (HIS genes) which expression is increased in migrating cells compared to the other tumor cells. We have demonstrated that the expression of six HIS metastasis-related genes (*ARHGDIB*, *CDC42*, *CAZPA2*, *XRCC5, PHACTR2* and *CAV1*) is decreased in the OCT-1-knockdown MDA-MB231 cells. These genes regulate migration and invasion of tumor cells.

The *ARHGDIB* (Rho GDP Dissociation Inhibitor Beta) gene regulates actin cytoskeleton reorganization mediated by the Rho family members. ARHGDIB overexpression in tumor cells is associated with tumor growth and metastasis in different cancers and positively correlates with tumor size, lymph node metastasis, and TNM stage in breast cancer patients. Moreover, ARHGDIB depletion leads to decreased proliferation, migration, and invasion of breast cancer cells. High ARHGDIB expression is the indicator of poor prognosis in breast cancer patients [42]. The *CAPZA2* (Capping Actin Protein of Muccle Z-line Subunit Alpha 2) gene encodes the F-actin capping protein. It binds to rapidly growing actin filament termini in the Ca-2+-independent way and regulates actin cytoskeleton and cancer cell invasion, which is tightly associated with cancer progression. The small GTPase CDC42 (Cell Division Cycle 42) promotes breast cell migration and regulates the signalling pathways which control diverse cellular functions including cell morphology, migration and cell cycle progression [43]. High expression of XRCC5 (X-ray Repair Cross Complementing 5) is associated with metastasis through the Wnt signalling pathway and predicts poor prognosis in patients with hepatocellular carcinoma. *PHACTR2* (Phosphatase and Actin Regulator 2) is significantly associated with lung adenocarcinoma and is one of the hub genes involved in esophageal squamous cell carcinoma. XRCC5 overexpression makes cancer cells resistant to standard chemotherapy [44]. CAV1 (Caveolin 1) is a multifunctional membrane protein that promotes migration and invasion of tumor cells and is a multifaceted driver of breast cancer progression [45].

We have demonstrated here that total OCT-1 and its OCT-1A isoform knockdown decreases MDA-MB231 tumor cell migration rate by 80% while exerting no statistically significant effect on cell proliferation. Our findings, therefore, suggest for the first time that a decrease in the cellular Oct-1 levels in triple-negative breast tumors may play a role at a metastasis stage and affects metastatic progression rather that primary tumor growth. It should be noted that the suppression of OCT-1 expression in the poorly invasive metastasizing triple-positive (estrogen receptor (ER)+, progesterone receptor (PR)+, HER2+) breast cancer MCF-7 cell line inhibits cell proliferation [46].

It has been previously demonstrated that growth and invasion properties of metastatic breast tumor cells in vitro may not correlate with each other and may be oppositely regulated by the Arg/Abl2 nonreceptor kinase acting as a switcher between the cell decisions to “grow” or to “go” [37]. During active migration and invasion, migrating tumor cells exhibit gene expression profiles similar to those which are typical of cells during embryogenesis when cell migration is required for normal morphogenesis. Apparently, the genes involved in cells division become switched off at that time since active proliferation starts only after tumor cell has settled itself in the target organ. 

It should be noted that in the course of embryonic cell differentiation the expression levels of certain OCT-1 isoforms in the limited size cell populations may be very high, which, however, does not result in their malignization [2,3,47]. The increase in OCT-1 levels itself does not inevitably cause malignization. It is rather that high OCT-1 expression resulting in OCT-1 production in wrong quantities in wrong cells at a wrong time increases the risks of malignization, promotes malignization, and makes it more aggressive. OCT-1 overexpression does not appear to be the trigger of cell malignant transformation. It is more probable that the increase in OCT-1 concentrations in already transformed cells (whatever the cause of malignant transformation may be) makes the tumor more malignant and more resistant to drugs and accelerates disease progression.

Hypoxia is often associated with malignant tumors making them resistant to antiblastic therapy [48]. Cancer cells have a strong need for energy to support their increased metabolism rates [21]. The Warburg effect, or the ability to produce energy predominantly by means of extremely intensive glycolysis with lactate production in the absence of oxygen, is characteristic of tumor cells. Glycolysis levels in the cells of a rapidly growing tumor may be by 200 times higher than those in the normal tissues, which considerably improves their resistance to and the ability to survive under “severe” hypoxia conditions. We and other authors have previously demonstrated that the increase in OCT-1 expression in the cells leads to the Warburg effect [2]. We have demonstrated here that decreased OCT-1 levels in breast cancer cells promote cell death under severe hypoxia conditions, potentially making the cells with decreased OCT-1 levels less viable during tumor growth and more susceptible to chemotherapeutics.

EPR stress develops in response to many different cellular stresses and induces, in its turn, the unfolded protein response (UPR), which represents a cascade of adaptive pathways directed at maintaining cell homeostasis and normal EPR function. The adaptive (protective) UPR is controlled by the three key proteins, PERK, ATF6, and IRE1. The depletion of ATF6 and IRE1 pathways results in decompensated EPR stress eventually leading to cell death [29]. We have shown here that a decrease in the OCT-1 concentration in breast cancer cells makes the ATF6 adaptive pathway less efficient thereby attenuating the adaptive response of cancer cells to EPR stress and promoting decompensated EPR stress and cell death.

Most chemotherapeutics cause EPR stress, while high resistance of cancer cells to this type of stress strongly reduces their sensitivity to chemotherapy. We have shown that a decrease in the OCT-1 levels enhances doxorubicin-induced breast cancer MDA-MB-231 cell death, or in other words, reduces cell resistance to chemotherapeutics.

The results reported here were obtained using the triple-negative breast cancer cell line MDA-MB231 (estrogen receptor (ER)-, progesterone receptor (PR)-, HER2-). We see it as the limitation of our study and cannot completely exclude the possibility that our findings hold more relevance for triple-negative breast cancers than for other cancer types. However, we believe that the data obtained in the present study indicate that selective OCT-1 suppression in human tumor cells may have a significant therapeutic effect and improve disease prognosis in breast cancer patients.

## Data Availability

Data is contained within the article.

## Supplementary Materials

The following supporting information can be downloaded at: https://www.mdpi.com/article/10.3390/life12091435/s1, Figure S1: Analysis of changes in OCT-1 protein and mRNA upon knockdown; Figure S2: The OCT-1 knockdown and apoptosis induction in MDA-MB231 cells.
