# Supplementary material for: Suppression of OCT-1 in Metastatic Breast Cancer Cells Reduces Tumor Metastatic Potential, Hypoxia Resistance, and Drug Resistance
Source: Life (Basel). 2022 Sep 15;12(9):1435. doi: 10.3390/life12091435 (PMC9502003; doi:10.3390/life12091435)
Supplement: Supplementary file 1 [file life-12-01435-s001.zip › life-1836067-supplementary.pdf]

Supplemental Materials

Suppression of OCT-1 in metastatic breast cancer cells reduces tumor metastatic potential, hypoxia resistance, and drug resistance

Alexander G. Stepchenko, Elizaveta V. Bulavkina, Tatiana N. Portseva, Sofia G. Georgieva, and Elizaveta V. Pankratova

Figure S1. Analysis of changes in OCT-1 protein and mRNA expression upon knockdown.

a

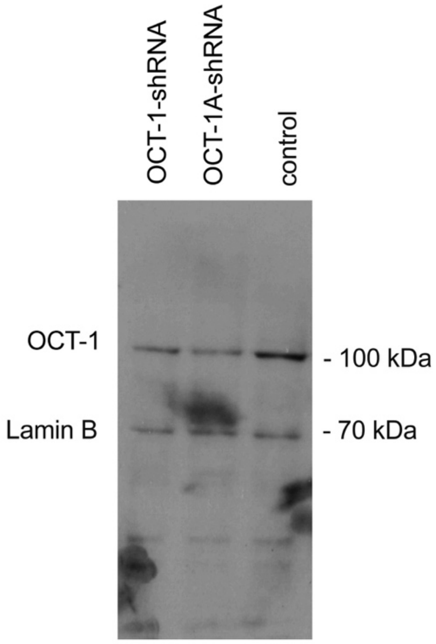

b

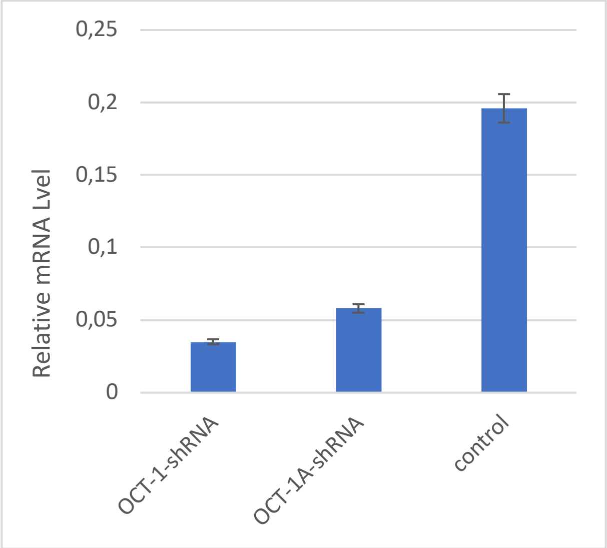

(a) Western-blot. OCT-1 (~100 kDa), Lamin B (68 kDa). OCT-1-shRNA – total Oct-1 knockdown, OCT-1A-shRNA – isoform Oct-1A knockdown.
(b) Real-Time PCR analysis. The relative OCT-1 mRNA level in the OCT-1 knock-down and control MDA-MB231 cells.

Oct-1 protein level.
Densitometry (reading/intensity ratio of each band).

	OCT-1-shRNA	OCT-1A-shRNA	control
OCT-1	0.17	0.185	1.0
Lamin B	0.96	0.98	1.0
ratio	0.177	0.18	1

Figure S2. The OCT-1 knockdown and apoptosis induction in MDA-MB231 cells.

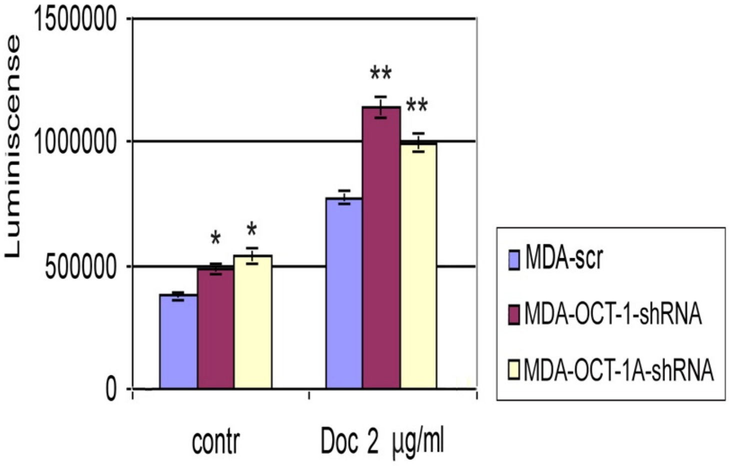

Determination of apoptosis by Bioluminescence caspase 3/7 assay (Promega)

The effect of OCT-1 knockdown on the level of apoptosis in MDA-MB231 cells: MDA-scr - cells transduced with the control "empty" lentivirus construct; MDA-OCT-1-shRNA - cells transduced with the lentivirus construct encoding the shRNA targeting total OCT-1 (POU2F1) protein; MDA-OCT-1A-shRNA - cells transduced with the lentivirus construct encoding the shRNA targeting the Oct-1A isoform.

Apoptosis was verified by Bioluminescence caspase 3/7 assay (Promega). MDA-MB231 cells were plated at 20 000 cells/well in 96-well plates in the final media volume of 100 µl. and treated with docetaxel (2 µg/ml) for 18 h or left untreated (control). Apoptosis was measured by a bioluminescent caspase assay (Promega). Caspase-Glo-3/7 Reagent was added to the wells and incubated at room temperature for 60 min. The luminescence of each sample was measured in a plate-reading luminometer as directed by the luminometer manufacturer. Results were plotted as signal-to-noise ratios. Background readings were determined from wells containing culture medium without cells. The graphs show means ± S.E.M. of three independent experiments. *t*-tests have been performed to compare the means. The asteriks indicate a *P* value relative to the control MDA-MB231 cells value. ($^{*}P < 0.05$, and $^{**}P < 0.01$).
